# Supplementary material for: Identification and Validation of an Aspergillus nidulans Secondary Metabolite Derivative as an Inhibitor of the Musashi-RNA Interaction
Source: Cancers (Basel). 2020 Aug 8;12(8):2221. doi: 10.3390/cancers12082221 (PMC7463734; doi:10.3390/cancers12082221)
Supplement: Supplementary file 1 [file cancers-12-02221-s001.pdf]

cancers

Supplementary Materials: Identification and Validation of an Aspergillus nidulans Secondary Metabolite Derivative as an Inhibitor of the Musashi-RNA interaction

Lan Lan, Jiajun Liu, Minli Xing, Amber R. Smith, Jinan Wang, Xiaoqing Wu, Carl Appelman, Ke Li, Anuradha Roy, Ragul Gowthaman, John Karanicolas, Amber D. Somoza, Clay C. C. Wang, Yinglong Miao, Roberto De Guzman, Berl R. Oakley, Kristi L. Neufeld, and Liang Xu

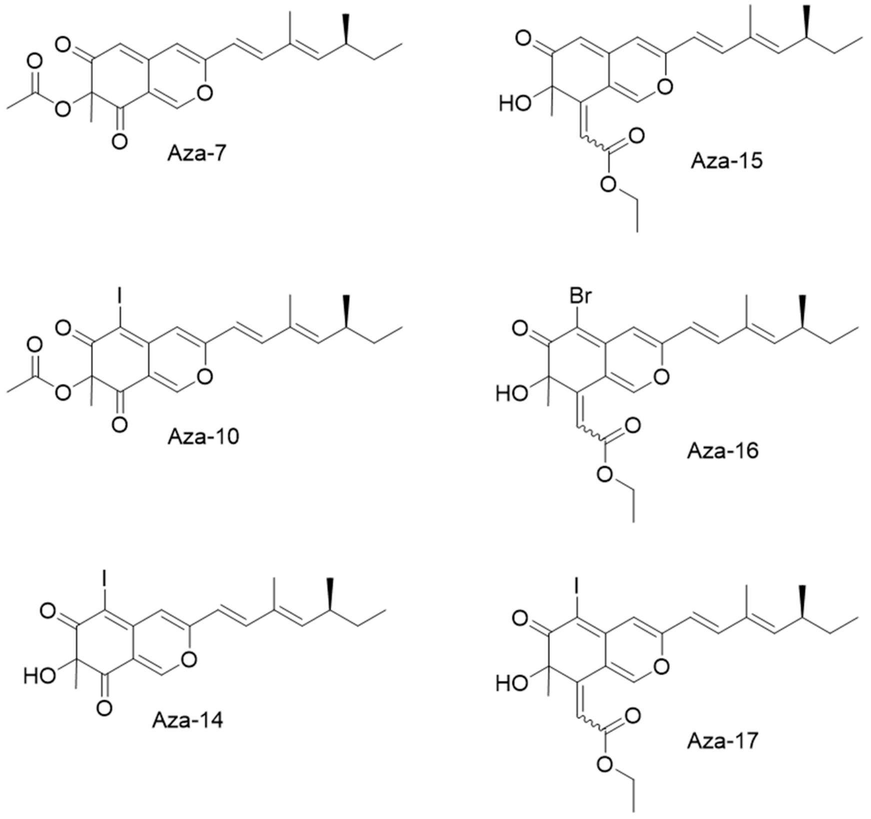

Figure S1. Structures of other azaphilones in the screening.

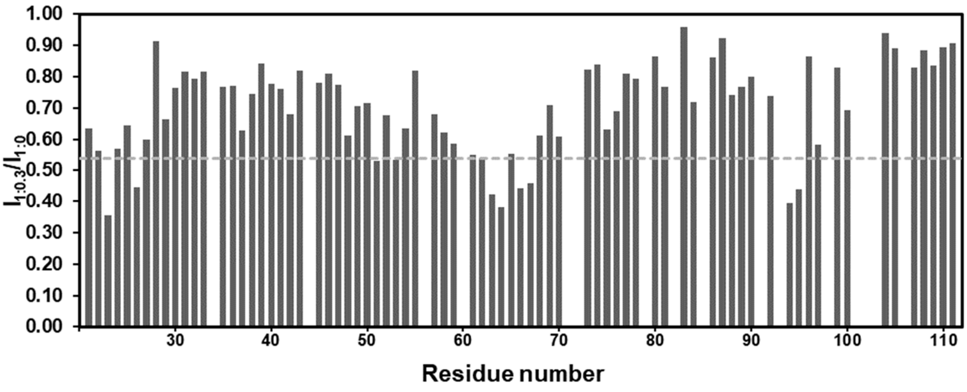

Figure S2. Plot of relative peak intensity for all non-overlapping MSI2-RRM1 resonances in the ligand bound form versus free state (I1:0.3/I1:0). Gray dashed line corresponds one standard deviation from the mean.

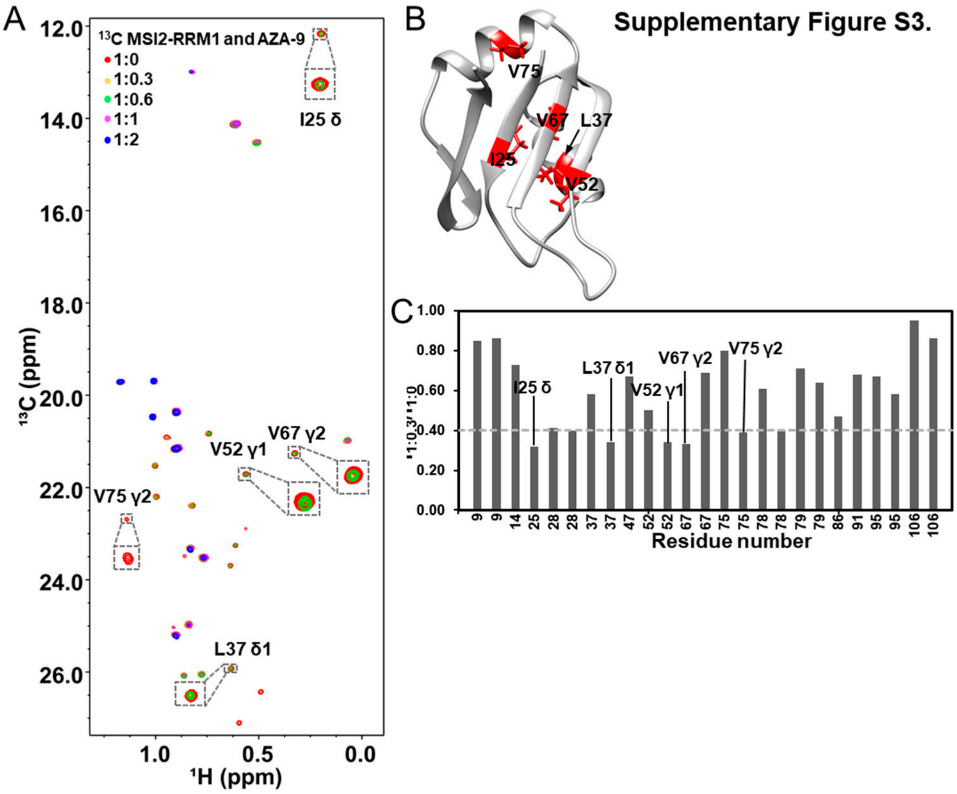

Figure S3. Aza-9 titration against ^{13}C ILV methyl labeled MSI2-RRM1. A) ^{1}H-^{13}C HSQC spectra of 80 μM ^{15}N and ^{13}C ILV methyl labeled MSI2-RRM with increasing molar ratios of AZA-9. B) Residues with peak intensity ratio lower than 0.40 are colored red. C) Plot of relative peak intensity for all non-overlapping MSI2-RRM1 ILV methyl resonances in the ligand bound form versus free state (I1:0.3/I1:0). Gray dashed line corresponds one standard deviation from the mean.

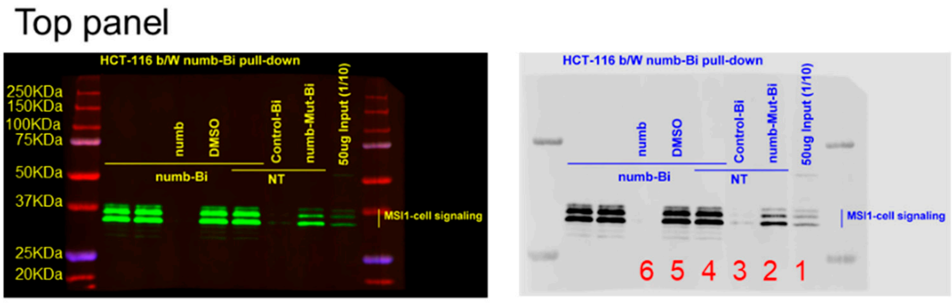

	6	5	4	3	2	1
Intensity	0.016	10.2	9.89	0.171	3.29	1.07
ratio	0	1	0.97	0.017	0.32	0.105

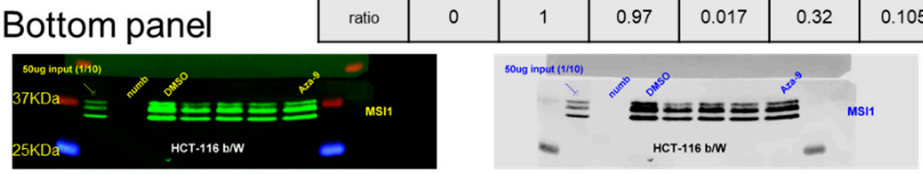

	INPUT	numb	DMSO	Aza-9
Intensity	1.15	-0.01	6.91	5.1
ratio	0.17	0	1	0.74

Figure S4. Figure 5B Supplemental.

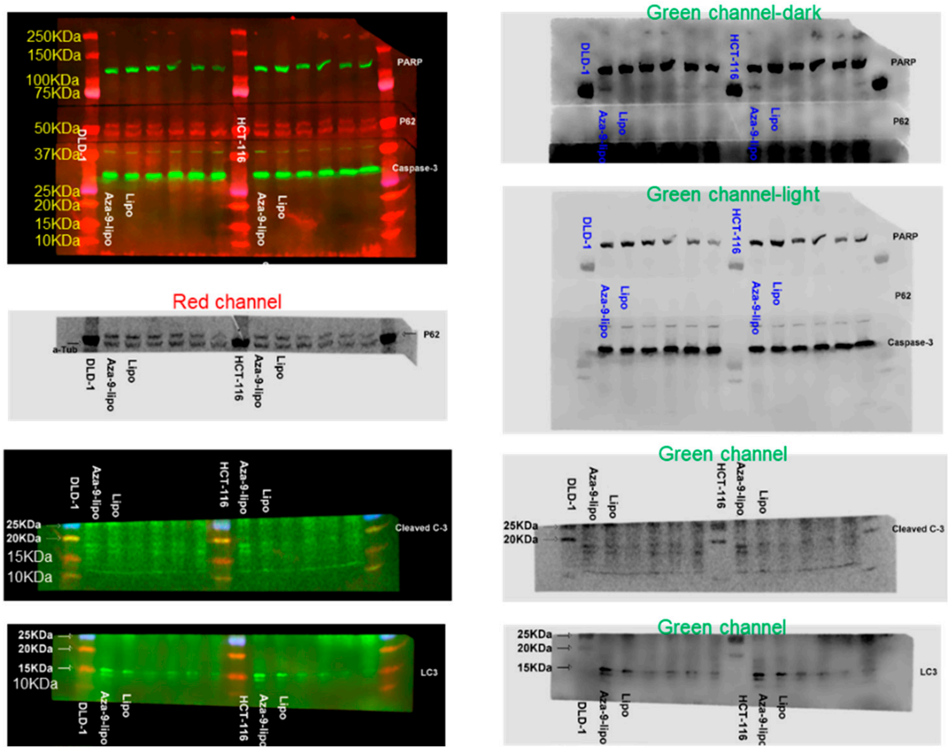

Figure S5. Figure 8B Supplemental.

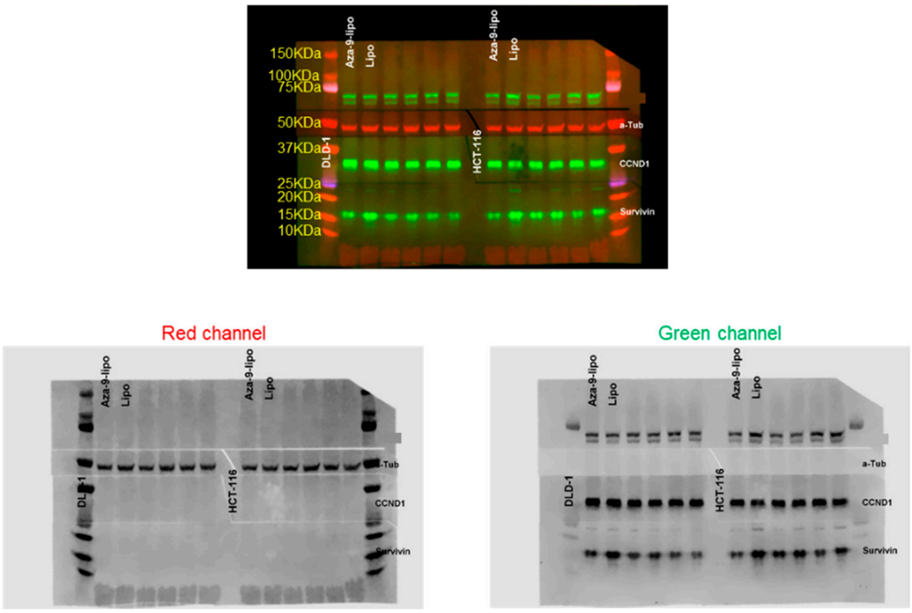

Figure S6. Figure 9B left panel Supplemental.

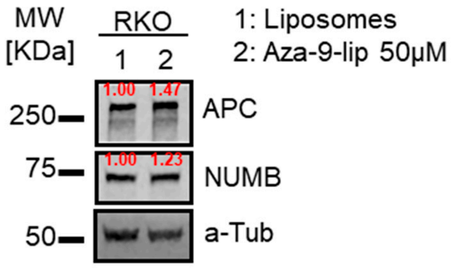

Figure S7. MSI direct targets APC and NUMB protein levels increased in RKO cells with Aza-9-liposome treatment.

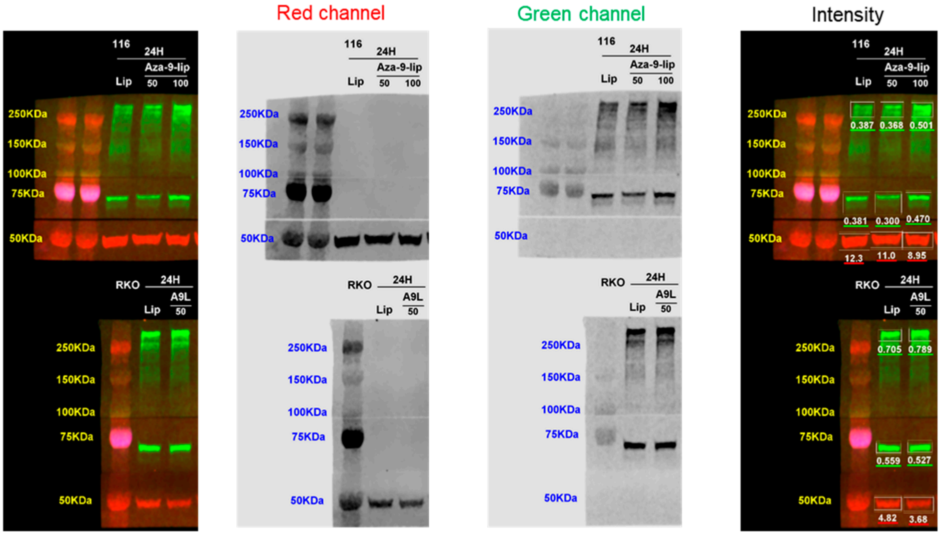

Figure S8. Figure 9B right panel and Figure S7 Supplemental.

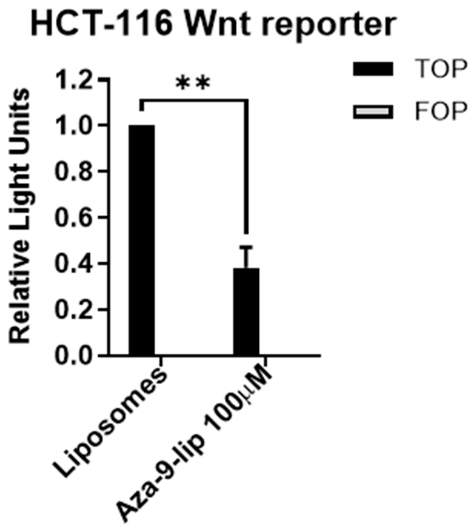

Figure S9. Aza-9-lip inhibits Wnt/β-catenin reporter. HCT-116 cells were transfected with TOPflash or FOPflash reporter constructs. Cells were treated with Aza-9-lip or liposomes only for 24 h in the presence of 20 mM LiCl.

© 2020 by the authors. Licensee MDPI, Basel, Switzerland. This article is an open access article distributed under the terms and conditions of the Creative Commons Attribution (CC BY) license (http://creativecommons.org/licenses/by/4.0/).
